# Mechanisms to explain the overshoot in bone remodeling markers after denosumab discontinuation: are we there yet?

**DOI:** 10.1093/jbmr/zjaf007

**Published:** 2025-01-23

**Authors:** Sabashini K Ramchand, Michelle M McDonald

**Affiliations:** Endocrinology and Metabolism Institute, Cleveland Clinic, Lerner College of Medicine - Case Western Reserve University, 9500 Euclid Ave, Cleveland, OH 44195, United States; School of Medical Science, Faculty of Medicine and Health, The University of Sydney, The Charles Perkins Centre D17, John Hopkins Drive, Australia

**Keywords:** Osteoporosis, diseases and disorders of/related to bone, Antiresorptives, therapeutics, Osteoclasts, cell/tissue signaling - Paracrine Pathways, Osteocytes, cells of bone

Denosumab, a fully human monoclonal antibody that binds to and inhibits the receptor activator of nuclear factor kappa-B ligand (RANKL), produces almost complete and sustained suppression of bone remodeling.^1^ In postmenopausal women with osteoporosis, denosumab 60-mg administered as a subcutaneous injection every 6 months continuously increases bone mineral density (BMD) and reduces vertebral, hip, and non-vertebral fracture rates.^1^ However, following cessation of denosumab (i.e. 6 months after the last injection), there is a rapid reversal of its inhibition on bone remodeling, with a transient overshoot of serum bone remodeling markers that exceeds pre-treatment levels.^2^ Serum bone remodeling markers peak between 3-6 months post-discontinuation and then gradually return to pre-treatment levels within 12-24 months.^2^ This phenomenon is associated with a decrease in spine and hip BMD and an increased risk of multiple vertebral fractures.^2^^,^^3^

The cellular and molecular mechanisms driving the overshoot in bone remodeling after discontinuing denosumab are not yet established. Our current understanding is based on observations from pre-clinical studies conducted in mice and cross-sectional clinical studies with small sample sizes and/or without adequate control groups, emphasizing the need for appropriately designed confirmatory clinical studies. Combined, these studies suggest that an enlarged pool of osteoclast precursors (OCPs) and/or recycled osteoclasts (“osteomorphs”)^4–6^; a decreased number of osteoblasts^7^; and an increased RANKL:OPG (osteoprotegerin) ratio which leads to unrestricted RANKL activity,^7^^,^^8^ may contribute to the overshoot in bone remodeling observed with denosumab discontinuation.

It should be noted that the overshoot of bone remodeling markers observed after cessation of denosumab is not limited to this drug. A similar effect has been observed following discontinuation of odanacatib, a cathepsin K inhibitor,^9^ and estrogen replacement therapy.^10^ This suggests that there may be “denosumab-independent” mechanisms also at play that warrant further investigation.

In the current issue of the Journal, Schini et al report findings of the first human study to investigate the effects of denosumab on OCPs compared to untreated controls.^11^ In this cross-sectional pilot study, peripheral blood was collected in the non-fasting state from postmenopausal women treated with denosumab (*n* = 15, median duration of treatment 4 years, range 0.5 to 9 years) and age and sex matched untreated controls (*n* = 15). Fluorescent activated cell sorting (FACS) was then used to measure peripheral blood mononuclear cells in real-time. The authors report that the population of OCPs expressing CD14+/CD11b + cells was higher in patients treated with denosumab compared to untreated controls, while there was no significant between-group difference in OCPs expressing CD14+/M-CSFR+ or CD14+/TNFRII+.

Supportive evidence for the findings reported by Schini et al were observed in a cross-sectional study of 15 postmenopausal women with osteoporosis. This study reported increased circulating mRNA levels of RANK and Cathepsin K in the denosumab treated groups compared to the untreated group (*n* = 5), indicating an increase in circulating osteoclast lineage cells.^5^ However, in this study mRNA levels were measured after denosumab discontinuation, which differs from the study by Schini et al, where OCPs were measured during denosumab treatment. Although informative, the findings of both studies need to be interpreted with caution given the cross-sectional design and the small sample sizes. Furthermore, given these limitations, rigorous evaluation of the associations between circulating OCPs and denosumab duration or between the number of OCPs and bone remodeling markers, were not possible.

An important limitation when attempting to study circulating OCPs is the absence of standardized cell surface markers to specifically identify these cells. The panel used in the current study lacks specificity, as the CD14+/CD11b + phenotype identifies a monocyte population with multi-lineage potential, rather than a distinct OCP subset. Incorporating markers such as CD16 and/or RANK could provide greater clarity regarding the potential of these cells to differentiate into osteoclasts.^12^ A recent study by Kaneko et al observed a reduction in circulating CD14 + RANK^hi^ OCPs in patients undergoing denosumab treatment, presenting a conflicting result.^12^ This discrepancy may be largely attributable to the differing cell surface markers used to define OCPs across the studies. The cross-sectional design of both studies also precluded assessment of temporal changes in circulating OCP numbers relative to the timing of denosumab administration. Defining temporal patterns in OCP numbers may be useful in establishing the optimal timing of sequential therapy approaches.

Circulating factors and cell populations do not always correlate well with local bone marrow levels. The results reported by Schini et al are limited to assessment of circulating cell populations and bone remodeling markers. Nevertheless, the observed increase in circulating osteoclast precursors with denosumab treatment is supported by pre-clinical studies that have investigated both local and systemic effects. In mice, RANKL inhibition led to the accumulation of both OCPs and osteomorphs,^6^ the latter arising from osteoclast fission, in the bone marrow. Notably, systemic RANKL and local RANKL mRNA levels were significantly elevated,^8^ while local OPG production was reduced following RANKL inhibition.^7^ Future clinical investigations are needed to determine whether the increase in circulating OCPs is associated with other circulating factors which are implicated in rebound bone loss, such as RANKL. Local assessment of OCPs and RANKL:OPG levels in bone marrow through bone biopsy studies and immunohistochemistry would also provide valuable mechanistic and spatial insight. Lastly, future prospective studies should be designed to determine whether the changes in OCPs with RANKL inhibition are transient, or persist into the rebound phase, and whether they are impacted by treatment duration.

Although the study by Schini et al provides the first human data to support pre-clinical findings of increased OCPs with denosumab treatment, a more comprehensive evaluation of this and other underlying mechanisms in larger longitudinal clinical studies are needed. It is hoped that an improved understanding of these mechanisms will facilitate the development of targeted strategies to mitigate the rebound effect and avert negative consequences in patients discontinuing denosumab therapy.
